# “*Where Creator Has My Feet, There I Will Be Responsible*”: Place-Making in Urban Environments through Indigenous Food Sovereignty Initiatives

**DOI:** 10.3390/ijerph20115970

**Published:** 2023-05-27

**Authors:** Elisabeth Miltenburg, Hannah Tait Neufeld, Sarina Perchak, Dave Skene

**Affiliations:** 1 School of Public Health Sciences, University of Waterloo, Waterloo, ON N2L 3G1, Canada; hannah.neufeld@uwaterloo.ca; 2 Department of Family Relations and Applied Nutrition, University of Guelph, Guelph, ON N1G 2W1, Canada; sperchak@uoguelph.ca; 3 White Owl Native Ancestry Association, Kitchener, ON N2C 2H6, Canada; daveskene@gmail.com

**Keywords:** indigenous food sovereignty, community-based participatory research, place, urban environments, land access, place-making, urban Indigenous Peoples

## Abstract

There is a growing emergence of Indigenous Food Sovereignty (IFS) initiatives across urban centers within many regions of Canada. Urban Indigenous communities are leading these efforts to revitalize Indigenous foods and agricultural practices while promoting food security and increasing Land-based connections within cities. However, the socio-ecological environments within these urban contexts affect IFS initiatives in unique ways which have not been previously explored. This study addresses these gaps by drawing on qualitative interviews with seven urban Indigenous people leading IFS initiatives within Grand River Territory (situated within southern Ontario, Canada). Applying community-based participatory methods, this research explored how place impacts IFS initiatives within urban environments. Thematic analysis generated two overarching thematic categories: Land access, and place-making practices, revealing a bi-directional, dynamic interaction between place and urban IFS initiatives. Relationships with landowners, control of land, and external factors determined how Land was accessed in urban environments. Place-making practices involved fostering relationships with Land, upholding responsibilities, and cultivating Land-based knowledges. Therefore, IFS initiatives are impacted by Land access, but also facilitate place-making for urban Indigenous Peoples. These findings demonstrate pathways towards Indigenous self-determination and IFS within urban contexts, which can be applicable to other urban Indigenous communities.

## 1. Introduction

Indigenous worldviews and ontologies recognize Land as more than a fixed geographical and physical space, but a “spiritually infused place grounded in interconnected and interdependent relationships” [1] (p. 301). Land encompasses all aspects of the natural world, including the plants, animals, water, earth, air, as well as the historical and contemporary relationships with spirits and ancestors to geographic places [1,2,3]. Land is dynamic, alive, and thinking [1,4,5]. As Daigle [5] conveys, the Land is “an animate being, relative, food provider, and a teacher of law and governance to whom we are accountable” (p. 266). Land has been capitalized in other academic writing to acknowledge the spirit and agency of Land as a sovereign, sentient being [1,6]. In this paper, we use ‘land’ in the lowercase to describe a physical and geographic location, but the capitalization of ‘Land’ is used intentionally to indicate the broader notions that Land encompasses. 

Land and place can be seen as synonymous because Lands are the ‘place’ of a community, inseparable from the people, culture, and spiritual identity [7,8]. Place is a slightly more expansive term which encapsulates the agency and awareness of Land [1], evoking the intangible relationships within the social and physical environments. Within Indigenous communities, place can be understood as an interconnected web of relationships and kinship ties, including animal and plant nations along with the Land, spirits, and ancestors [9]. These relationships can be conceptualized as intimately connected with, even constitutive of, places themselves [10]. Place has meaning because of the agency that comes from the Land and is understood in multiple relational and historical ways for diverse peoples [4,5]. Daigle [5] offers that according to Omushkegowuk Cree ontologies, “place is shaped by local people, knowledge systems, and Land-based practices as well as by colonial-capitalist structures of power” (p. 268). These interconnected and interdependent relationships form a distinct identity, a personality of place that can be related to and known [11]. Place is dynamic, emergent and determines something of human actions and social conditions [4,9]. There is a sense of responsibility that comes with knowing a place intimately and in relational ways. 

Land and place are central to the health and wellbeing of Indigenous communities [12,13,14,15]. Land provides sustenance and nourishment of body, heart, mind, and spirit through the acquisition and processing of food from Lands and waters [16]. Land is an inseparable aspect of Indigenous food systems, which are rich in ecological place-based knowledge reflecting the relationships which have evolved over generations forming a way of life that guides food practices [17,18,19]. Indigenous food systems reflect the importance of nurturing reciprocal relationships with the Land, plants and animals recognizing food is a gift from Creator; which instills responsibilities to sustain a healthy environment [16,18,20]. Responsibilities to the Land are apparent within Indigenous food systems as they keep individuals and communities healthy and connected [20,21,22].

Urban Indigenous populations have distinct health, social, and cultural needs which are impacted by place and space in unique ways [23,24]. Indigenous Peoples living in urban centers have experienced migration, movement, and displacement from their original homelands through processes of settler colonialism resulting in disconnection to Land, and loss of culture, knowledge, and identity [25,26,27]. The majority of Indigenous Peoples within Canada now live in urban centers [28] and Indigenous populations in cities continue to grow. Within the province of Ontario, 85.5 percent of First Nations, Métis and Inuit live off-reserve [28]. It is expected that urban Indigenous Peoples will reach ten percent of the population in Canada’s five major cities by 2031 [29]. As this segment of the population continues to grow, the social determinants of health must consider structural factors such as colonization, racism, loss of Land and culture, as well as patterns of urban migration [13]. The impacts of place on urban Indigenous health and well-being are complex and demonstrate the need for more research in this area [30].

Colonial policies have dispossessed Indigenous Peoples living in urban centers from the Land, disrupting Indigenous food systems directly and indirectly [23,27,31]. Canadian cities were deliberately built where Indigenous communities were established [32] displacing Indigenous Peoples to locations which often had less favorable agriculture conditions, access to resources and trade routes [33,34]. The formation of reserves during the 1800’s restricted access to territories, and thus the ability to acquire food sources [33]. The residential school system was intentionally created to sever intergenerational transfer of language, culture, and knowledge, impacting the ability to practice Indigenous foodways [12,27,35]. These processes that have reduced Indigenous Peoples’ access to Land and resources of their local environments, are referred to as environmental dispossession, which can result in the inability of affected populations to sustain, share, and practice Indigenous knowledges [12].

While the creation of reserves was intended to segregate Indigenous Peoples from settler cities [36], government policy agendas in the early 1900’s encouraged migration of individuals, families, and communities to urban centers to further assimilate Indigenous Peoples into settler society [37]. After the second world war, economic conditions worsened on reserves, and the increase in urbanization in cities influenced migration towards urban centers for employment and education opportunities [38]. The impacts of colonialism and assimilative policies associated with urbanization have eroded Indigenous knowledges, fractured families and relationships that have existed between Indigenous Peoples, within communities, and the Land [39]. These structural determinants have affected the health of communities, resulting in detrimental effects on physical and social wellbeing experienced by Indigenous Peoples living in urban environments.

The historical and ongoing processes of colonization have shaped urban food environments resulting in an increased prevalence of food insecurity among Indigenous Peoples living in urban centers [14]. The higher cost of living, lower incomes, limited transportation to grocery stores and more exposure to lower-quality marketed foods in urban environments make it challenging for urban Indigenous people to access preferred foods [23,25,40]. Indigenous Peoples living in cities often rely on social and family connections to their home territories to access Indigenous food sources, but being physically and socially disconnected from these territories restricts participation in Indigenous food systems [23,40,41]. Without a connection to their community, urban Indigenous Peoples face a loss of skills and knowledge necessary to acquire and process Indigenous foods [26]. While food sharing between urban-rural-remote networks supports access to cultural foods from outside the city, such as wild game [41,42], the ubiquity of store-bought, market-based foods predominate what is available and accessible to the Indigenous consumer [25]. Therefore, the ongoing impacts of colonization that restrict access to Land, and disrupt knowledge transfer surrounding Indigenous food practices, can result in more limited food skills and access within urban Indigenous communities. Compounded by the influence of western food systems in urban environments, Indigenous Peoples living in cities experience food insecurity not just from lack of income to afford to purchase food, but the dispossession and disruption of relationships with Land, knowledge and foodways. 

There is a growing body of literature documenting IFS initiatives emerging within urban environments. For example, the establishment of Indigenous food and medicine gardens at post-secondary institutions [43,44,45], the facilitation of Land-based programming at health centers [46], and the implementation of food skill workshops in partnership with urban Indigenous organizations [26,47]. This developing body of research explores how structural determinants and mechanisms of environmental dispossession affect urban Indigenous food systems [14,23,25,44,45,46,47,48]. This paper contributes to this growing knowledge base by exploring how place impacts urban IFS initiatives, examining the broader socio-ecological factors that affect urban food environments. This research study is part of a larger Canadian Institutes for Health Research (CIHR) funded project aiming to bridge spaces and places between on-reserve, urban and institutional environments to strengthen pathways towards IFS across southern Ontario. Engaging with participatory elements of community-based research and relational approaches of Indigenous research methodologies, this exploratory study examined how Indigenous-led food sovereignty initiatives contribute to place-making within four urban centers. The findings presented in this paper are part of a research project focusing on the urban component of the CIHR study. Complementary results from this work, describing the principles and practices of these IFS initiatives are published elsewhere [45,48]. 

## 2. Materials and Methods

### 2.1. Community Context

This research study took place within present day Waterloo-Wellington region, two municipalities located in southern Ontario, Canada. The region is home to more than 700,000 people who mostly live in the four major urban centers: Kitchener-Waterloo, Guelph, and Cambridge. These urban centers are surrounded by agricultural areas and rural communities; all of which are connected by the Grand River or its tributaries [49].

The Waterloo-Wellington region is part of Treaty 3, the territory of the Mississauga’s of the Credit. The cities of Kitchener-Waterloo and Cambridge are located within the Haldimand Tract, which encompasses six miles on either side of the Grand River-Land promised to the Six Nations of the Grand River as allies during the American Revolution [50]. Prior to the arrival of European settlers, Attawandron (Neutral) villages existed along the Grand River, demonstrating the significance of this area for migration and trade. The Dish with One Spoon wampum agreement also governs this territory, which establishes principles to share the Land: to take only what you need, leave some for the next person, and to keep the dish clean [50]. This agreement initially formed between the Anishinaabe and Haudenosaunee to share hunting grounds and resources between the Great Lakes region, and then included Europeans upon their arrival to this territory [51].

The 2016 census data shows that 12,370 individuals self-identify as Indigenous in the Waterloo-Wellington region, representing 1.5 per cent of the total population [28], however, census counts often underestimate urban Indigenous populations two to four times [52]. Local estimates project up to 22,000 individuals identify as Indigenous within Waterloo region [53]; 62 per cent of whom are First Nations, 35 per cent are Métis and 3 per cent are Inuit [28]. The Indigenous population in the region has increased in the last several decades because of a growing number of youth and children, reflecting a similar trend in many urban centers across Canada [53]. There is continual change and migration within the local Indigenous community, as the majority have moved to the region for employment, education, or family, while only 15 per cent of people have lived in the community for their entire life [54]. Currently, there are several Indigenous organizations and grassroot community initiatives supporting the local Indigenous community. Of these groups, some are working towards Indigenous food sovereignty (IFS) or promoting Indigenous food security in the region, four of which were included in this study.

### 2.2. Theoretical Frameworks and Research Process

This study applied a decolonizing approach to the research process by prioritizing Indigenous ways of knowing and employing continual reflexivity of relationships, actions, and experiences through relationship building with Indigenous community [10,55,56]. Indigenous research methodologies aim to decolonize power differences in the research process to produce knowledge through collaborative, relational, non-hierarchical approaches [56,57]. Indigenous research methodologies recommend the use of community-based participatory research (CBPR) to center the relational aspect of research with Indigenous communities [58,59]. The participatory nature of CBPR complemented the relational, reciprocal, and action-oriented nature of IFS [18] creating spaces for relationship with Land and community while engaging in Land and food-based activities which informed the research.

The participatory element of this research was important for the first author (E.M.) to understand the community context and become aware of the IFS activities taking place in the region. As a non-Indigenous graduate student new to the community, E.M. attended community events and became involved with gardening activities to build relationships with Indigenous peoples leading IFS initiatives. She relied on the pre-existing research partnership held by her supervisor (H.N) to connect with Wisahkotewinowak, an urban Indigenous Collective growing Land-based relationships through garden spaces and sharing Indigenous foods within the region [60]. E.M. initially joined the Collective as a research assistant to coordinate meetings, food workshops and events, through which she built relationships with others in the Collective including the third (S.P.) and fourth authors (D.S.). This process of engagement helped to identify a research question that would be beneficial to the Collective and other IFS initiatives in the urban community.

The USAI Research Framework developed by the Ontario Federation of Indigenous Friendship Centres (OFIFC) guided the research process [61]. The USAI Research Framework is governed by four ethical principles to be applied when research is conducted with urban Indigenous communities: Utility, Self-voicing, Access, and Inter-relationality. The participatory nature of this research fostered inter-relationality and ensured the utility and accessibility of the knowledge produced. A research advisory committee was established with institutional and community partners, of whom some were also research participants. The committee provided a space for findings and updates to be shared, allowing for research participants and project partners to have an active voice in the research process. The results were also shared with individual participants to ensure accuracy and invite feedback on how their voices were presented before any data was published. Centering voices from the urban Indigenous community throughout the research process recognizes local knowledges as accessible, authentic, and inherently valid. Therefore, applying CBPR and the USAI principles demonstrates an effective approach to decolonize the research process [59,61]. 

### 2.3. Data Collection and Analysis

Participants were recruited to this study through pre-existing relationships held as part of the Wisahkotewinowak Collective. The community-based aspect of this research helped identify participants who were engaged in local IFS initiatives, self-identified as Indigenous and lived in the urban centers of Kitchener-Waterloo, Guelph, or Cambridge, Ontario. Using purposive and snowball sampling strategies other organizations and initiatives were recruited to identify potential participants. Information letters and consent forms were sent by email to individuals that expressed interest in participating in the research. Participants were offered asemma (tobacco) at the time of the interview, to affirm the intentionality of the conversation and respect cultural protocols acknowledged by the local Indigenous community when knowledge is shared [62]. 

A semi-structured interview guide was developed with input from the research advisory committee. Participants were asked about their engagement in IFS initiatives, perspectives on Indigenous food access in urban centers, and how the urban environment impacts IFS initiatives. Some examples of interview questions included: can you tell me about the food related work you are involved in? What are the goals of this initiative? How do you think this work supports Indigenous food sovereignty? How do you think where you live affects your relationship to Indigenous foods? What do you think is unique about being involved in the work you do in an urban setting? What challenges have you experienced implementing this initiative? What suggestions do you have that would further promote participation in local food practices? Semi-structured interviews took place in-person, which lasted between one and two hours and were audio recorded, except for one participant who chose to not be recorded. Instead, this participant typed out responses to interview questions after the interview was held and this document was treated as the transcript for analysis. Audio-recorded interviews were transcribed verbatim, then shared with participants as a form of member checking, allowing participants to edit quotations so their words were accurately reflected, and any unwanted identifiable details could be removed. At the request of participants, additional slight edits were made to quotations included in publications to improve readability. 

Thematic analysis was completed using NVivo version 12 for Mac (Denver, Colorado, USA) to identify patterns in the data [63]. The lead author also documented field notes in a personal journal to glean insight and reflect on assumptions and biases during the research process as a form of reflexive analysis [56,64]. Preliminary themes were shared with the research advisory committee and interested participants for feedback before results were finalized. Participants received a $50 grocery gift card as an honorarium for their participation. Data collection occurred between August and November 2020. Ethical approval was received from the University of Guelph Research Ethics Board (REB#20-02-021). 

## 3. Results

### 3.1. Details of Urban IFS Initiatives

Seven participants belonging to four urban IFS initiatives in the Grand River Territory participated in this study including the Wisahkotewinowak Collective, Waterloo Region Indigenous Food Sovereignty Collective, Waterloo Indigenous Student Centre – Shatitsirótha, and the North End Harvest Market. Participants discussed how IFS initiatives operated and provided details on garden locations, Land access arrangements, and activities performed. These details are summarized in Table 1, showing participants’ connection to the respective IFS initiative. 

The Wisahkotewinowak Collective operated four gardens at the time this research was conducted (summer 2020): a small Tea Garden located at the University of Guelph Arboretum, a produce garden located at the University of Waterloo Environmental Reserve, a teaching garden at the Blair Outdoor Education Centre, and a Three Sisters garden, located at Steckle Heritage Farm, a non-profit farm in Kitchener, Ontario. The foods and medicines harvested from the gardens were shared with local post-secondary Indigenous student centers, food preserving workshops, and through a food share program in partnership with White Owl Native Ancestry Association (WONAA), a local Indigenous agency. 

The Waterloo Region Indigenous Food Sovereignty Collective began in 2020 as a response to the food insecurity needs at the onset of the pandemic. The Waterloo Region IFS Collective applied a grassroots neighborhood approach of food sharing and meal making to provide for community. They had 17 backyard gardeners who grew food for the collective, and maintained one larger seed garden with corn, beans and squash that was on private Land gifted for the community to use for the season. The University of Waterloo Indigenous Student Centre (WISC), named Shatitsirótha’ meaning, to ignite the flame, oversaw a small produce garden, and medicine gardens at the ceremonial fire site on campus. The campus garden grew enough food that contributed to the WISC fall feast and soup and bannock lunches held weekly at the Centre. The North End Harvest Market provided culturally significant foods to the local Indigenous community by sourcing foods from various Indigenous suppliers, but at the time did not have direct Land access for food growing purposes.

### 3.2. Interactions between Place and IFS Initiatives

**Figure 1:**
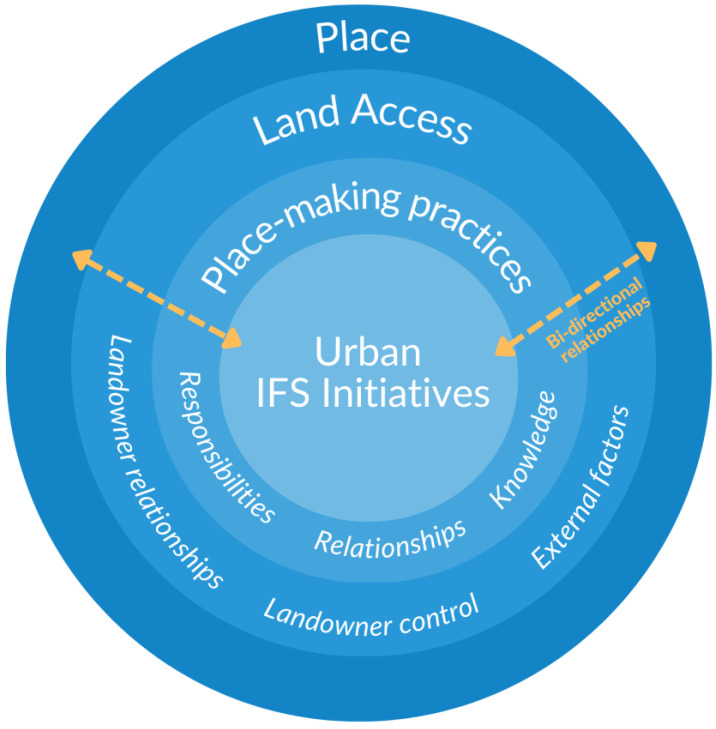
Model demonstrating Land access and place-making practices as dynamic interactions between urban IFS initiatives and place.

While each initiative operated in distinct ways, they all engaged in similar Land- and food-based practices and followed principles around relationality, responsibility, and reciprocity (see [45]). Land was a central theme across participant interviews and was discussed in relation to IFS initiatives as both a space to access in order to grow food, and a place to build relationship with. Thematic analysis of the interviews identified two overarching categories: Land access and place-making practices. The themes pertaining to Land access include relationships with landowners, landowner control, and external factors. Themes relating to place-making practices are Land-based responsibilities, relationships, and knowledge (see Table 2). The relationship between these overarching themes, IFS initiatives and place are presented in Figure 1. 

The model conceptualizes a bi-directional, dynamic interaction between place and urban IFS initiatives through Land access and place-making. These interactions can be interpreted by reading the model from the outer layer inwards, and conversely, from the center, outwards. This is depicted by the bi-directional arrows moving between and among each component of the model. Land access is a necessary condition for Land-based activities to occur, therefore, the influence of landowner relationships, landowner control and external factors impact how IFS initiatives are taken up in urban environments. These processes are demonstrated in the model moving from the outer layer inwards, depicting how structural determinants in the broader environment shape how IFS initiatives access Land. Moving from the center outwards, IFS initiatives provide the conduit for place-making practices to occur, generating access and connection to Land through relationship, responsibility, and knowledge formation. These practices materialize the interconnected web of relations in the environment constitutive of place. Place, therefore, shapes and is shaped by these dynamic interactions influenced by IFS initiatives. The interactions among these concepts are not consistent in every circumstance, however, because relationships are never static, but rather continue to evolve and change in various contexts. These interactions will be illustrated through the individual themes to follow and further explored in the discussion section. 

### 3.3. Land Access

#### 3.3.1. Relationships with Landowners

All participants spoke repeatedly about access to Land as a significant factor that impacted IFS initiatives, because ultimately Land is required for IFS initiatives to grow food. Relationships with landowners were necessary to access Land, but the nature of these relationships determined whether experiences were positive or more challenging. Beth shared how a request for Land for their group was met by a private landowner who offered an acre of Land for their use. Access to this space was beneficial because of how relationships were formed around the Land, clearly establishing the purpose of the garden was for the community, who held collective responsibility of the space.

Pre-existing relationships helped participants gain access to Land to pursue IFS initiatives. Dave explained how one of the members of the Wisahkotewinowak Collective was also a board member at a non-profit heritage farm in Kitchener. This connection enabled a garden to be established at this site soon after the opportunity was discussed, as the non-profit organization had this internal support. The history and structure of Steckle Heritage Farm as an educational farm aligned with creating space for an Indigenous garden, which may likely have provided support for this arrangement [55]. This initial connection in 2017 has allowed Wisahkotewinowak to further their partnership with the non-profit and secure access to this space from year to year. While this demonstrates an example of a supportive relationship for an urban IFS initiative, these experiences are dependent on the relationships with the staff and board members who still ultimately determine how the Land is managed and used. 

#### 3.3.2. Landowner Control

Participants also described challenges accessing Land, particularly the barriers around navigating relationships with institutions or governing bodies that ultimately act as gatekeepers of the Land. Dave also shared his experiences accessing Land for another garden at the University of Waterloo Environmental Reserve as more challenging, simply because there was no existing relationship to that place, and it was unclear who to talk to. He explained: “[I]t took three years of asking questions and stuff like that before we were able to get [access to that land].” There remains uncertainty about the longevity of access to this space simply because there is not a clear relationship with the institution. 

Garrison and Rachel further described how Indigenous self-determination and sovereignty around Land use is challenged when landowners have the ultimate control over the Land, even if they provide access for Indigenous community use. Garrison explained how this was a barrier to implementing IFS initiatives in urban environments:

*The biggest barrier to food sovereignty for Indigenous people in urban centers is control of land. We don’t own land in the western, in the Canadian legal system, so if you don’t own land then we’re just borrowing land to grow food on, or to hunt food on. So, you can’t be sovereign, right? Because at any point in time… that land can be ripped away from you.*

Garrison further explained that: “most ownership is not in the hands of Indigenous people and even in urban centers where there is park land or there is charitable trust land, they also don’t have control over what happens there.” Rachel also spoke about how accessing Land was a challenge when other groups were in control of the Land being used for IFS initiatives. She shared: “[W]e’ve been trying now since 2015 to get land, and three different venues that were being used that had long term plans created were then shuttered because the settlers decided to go a different way.” Rachel recognized the tensions between landowners, (in this instance, settlers) and local community goals. Therefore, in these instances when landowners provide access to Land but have ultimate control, there is a lack of Indigenous sovereignty and self-determination.

Considering the challenges regarding land ownership for the longevity of IFS initiatives, Rachel brought attention to the fact that urban IFS initiatives are taking place on treaty Lands. Reflecting on this, she raised a number of important questions such as: “who gets it? how is it maintained? how is it used? who was contacted? who was consulted?” Rachel considered that band councils and clan mothers of the specific treaty Lands as well as Elders in the local community should be informed. Additionally, Garrison considered how a governing system exists for First Nations communities living on reserve to determine how the Land is used, but within urban centers there is not an Indigenous-led governing system to provide input, nonetheless decision-making on how Land is used in the city. Both Garrison and Rachel recognized these processes as strenuous and a complex challenge to navigate, and may be more complicated in urban environments. 

#### 3.3.3. External Factors

While relationships with landowners and landowner control affect land access in urban centers, the physical and social environment also impacts IFS initiatives. One such challenge presented by Lori is the visibility of garden spaces and proximity to urban dwellers. For instance, the Shatitsirótha gardens are located in a semi-public and accessible area on the University of Waterloo campus, which poses a risk of other people interfering with the gardens. Lori described an unfortunate situation where the strawberries from the garden at the sacred fire site were intentionally damaged. She spoke about how this was frustrating because signs were in place to inform the public about the purpose of the gardens and to respect the space, but unfortunately, as she shared: “somebody ripped all our signs down and took all the strawberries.” She continued, explaining how this instance was particularly frustrating because these strawberries were going to be shared for use in ceremony. 

Therefore, external factors within the social, physical, and political environments impacted participants’ ability to access and use Land in urban spaces in self-determined ways. While physical access to Land is a necessary factor for the implementation of IFS initiatives, broader structural determinants must be considered to address long term Indigenous self-determination and sovereignty. Ultimately, participants demonstrated how IFS initiatives provide the pathway towards creating more supportive environments, by emphasizing responsibilities to the Land and strengthening Land-based relationships and knowledge within urban centers. 

### 3.4. Place-Making Practices

#### 3.4.1. Responsibilities

All participants described Land as a place where relationships are formed, knowledge is fostered, and responsibilities are learned. These themes are categorized as place-making practices because they demonstrate an engagement with Land, in a relational, spiritual, and emotional way. This perspective acknowledges the spirit and agency of Land which evokes an awareness of responsibility to the Land, who is a relative to love and care for. This stands in significant contrast to Euro-centric perspectives around legal rights and ownership of Land exhibited within Canada. Sarina talked about how legally owning Land may be necessary to manage the Land in an Indigenous determined way while living in the current system, but ownership does not posit control or rights to the Land. She suggested that access through ownership can help facilitate spiritual relationships and responsibilities to Land. Dave reflected on how this perspective of having responsibilities is unique to being Indigenous: “From an Indigenous perspective…we’re not rights driven, but responsibility driven… and understanding who you are is understanding your responsibilities, not understanding your rights”. 

This perspective of responsibility over rights was exhibited during the conversation shared with Beth. This interview was held outside on her porch, where the gardens were visible. This prompted a question from the interviewer: “So this is all of your land and things that you’ve planted in your backyard?” Beth offered a correction in her response: “It’s the land I’m responsible for. I don’t see it as mine.” This exchange demonstrates how an Indigenous worldview shapes Beth’s understanding of her responsibility, which is distinct from a western perspective of rights and ownership of Land. Her perspective offered that responsibility comes from a deeper relationship to Land. Curious to explore this further, the interviewer asked Beth about how she understands her responsibility for the Land around her. She summed up: “Well because I’m on it. And my feet are on it. So, where Creator has my feet, there I will be responsible”.

Access to Land ultimately helped participants fulfill responsibilities to the Land, which they recognized were part of their inherent relationships with Creator. Nookomis shared that knowing how to be responsible begins with the Anishinaabe Creation story. She explained how each of us, as human beings with our unique gift of choice, have a responsibility to acknowledge, honor, respect, sustain, and reciprocate all the gifts Created. Therefore, we each hold a sacred relationship with Creation that must be honored with humility and gratitude because we are all interconnected. To her, this personally meant, “being a seed keeper of a number of plants/roots/barks/flowers/seeds specific to my Clan. I do this in Ceremony, with Community to produce, gather, prepare, feast and for healing.” Garrison described how being in relationship with plants and seeds presented an invitation to fulfil and uphold responsibilities to Creation:

*Planting and saving seeds and planting again are these beautiful ways of really being able to witness the unfolding aspect of life. So, I was always really intrigued by that and really excited to be able to use my hands to co-create with, in relationship with these plants, but also in relationship with the Creator and what the Creator had intended for us to be, right? Cause these relationship beings, when we receive from those plants that we are also, that our responsibility is to save those seeds and keep that plant alive.*

Dave also described that being responsible for the Land extends to include all of community. He shared:

*We want to be responsible for the land and we want to be responsible for the community, right? Our understanding of community comes from the land, we’re rooted in the land and you can’t separate those two things. If we’re really responsible for the land, then we are responsible for the community as well.*

Access to Land therefore helps instill feelings of responsibility to the Land, even within urban centers. IFS initiatives help foster relationship to the Land, plants, more than humans and people in community. This relationship making to place was integral to honor responsibilities and also be a pathway to build Land-based knowledge. 

#### 3.4.2. Relationships

All participants demonstrated how they were able to establish relationships with Land in urban spaces, often through Land and food-based practices. IFS initiatives provided an opportunity for participants to strengthen their relationships with Land while honoring responsibilities in significant ways while living in urban environments. This was actualized by Land and food-based practices, most significant were seed saving, growing, and gathering food. Participants described how growing culturally significant plants from traditional seed varieties, such as corn, beans, and squash can create relationship with a place, recognizing the relational importance of Indigenous foods. Garrison explained how growing food and seeds that are significant to the Territory where he lives, and to his ancestral homelands, helped strengthen relationships and connection to place:

*For example, here the Mohawk white corn … has connection with the people of this territory. I am also growing blue corn … [that], has an even longer historically you know, time wise, connection to Anishinaabe people here in this territory… I am growing Lenape squash, which is from my people from the East Coast, so there is still a connection for me to my people and the land where I, where my family comes from, but that is still brought here and talked about in relationship to, with me and this place, in the urban centre.*

This was significant for Garrison recognizing that migration and movement displaced Indigenous peoples from their homelands historically, but those foods are often the connections to those places. Beth also recognized the significance of growing white corn from Six Nations in the gardens located within the Haldimand Tract, stating: “the granting of the land in this Territory to [The Haudenosaunee], which we all know has not been fulfilled, but just doing my best to honor their traditions and their seeds on the land.” She offered that growing this corn was out of respect for Haudenosaunee peoples in the community since she was Anishinaabe. Therefore, growing food provides an avenue for relationship building with the Land, which creates opportunity for learning and understanding of responsibilities to fulfil in the places we live. 

#### 3.4.3. Knowledge

An important aspect to upholding Land-based responsibilities is gaining an understanding of how to take care of the Land. Several participants recognized that building a relationship with Land provides a starting place for knowledge to flourish. Sarina spoke about how she relied on the Land to help her learn about taking care of the gardens when she first started being involved in the food sovereignty work. She shared:

*So, we’re able to communicate with the plants and understand what they like based on their soil and their water intake. And we’re learning more and more about what they need, and we give what we can, we put down tobacco, we give them water, and I’ve read somewhere that to take from a plant is also sustaining that’s plants needs because that’s what it is grown to do, right? And that’s how some plants continue to grow, they’re eaten and then disposed of elsewhere, and that’s how they germinate and grow. It is a mutually beneficial relationship*.

This point illustrates that knowledge of the Land can be fostered through building Land-based relationships. This was important to Dave and Beth, who recognized that many Indigenous people living and growing up in urban spaces feel like their Land-based knowledge is limited. Rachel remembered learning about foraging food and medicines from forest walks she went on with her grandpa and dad when she was younger. She explained:

*They would take me in the woods and show me ‘this is this, this how you use it, this one’s medicine’ but I didn’t really associate that as traditional food until we started doing the [food sovereignty] stuff ‘cause I just saw them as the forest walks I did with my dad and my grandpa*.

While this knowledge was learned at a young age, Rachel realized the importance of this Land-based knowledge after participating in community discussion groups around traditional foods, the cycle of the seasons and sustainable Land-based practices. It was only after being in community that spurred her memory, and she realized the importance of the knowledge she held. 

Beth pointed out that knowledge requires community, explaining that everyone holds a little bit of knowledge. Coming together in community helps strengthen those building blocks of knowledge to put together a more complete picture. This is significant in an urban setting where connections to knowledge holders and Elders are often more limited. However, the diversity of Indigenous people living in urban centers provides an opportunity to develop localized Land-based knowledge from wider perspectives. Beth spoke about how learning from one another in an urban setting is unique:

*[Indigenous people living on-reserve] have many benefits and blessings based on where they’re raised, and we have many benefits and blessings because of where we’re raised. So, I get access to so many different Nations and so many different Nations’ knowledge and so many different Nations traditions. Right? And it’s like I feel like I have a more complete understanding of who Creator is.*

Dave shared how engaging in food sovereignty work as a Collective was a way to build Land-based knowledge. He recognized the gardening efforts he was initiating were being done in a new way to strengthen Land-based relationships in the city. He wanted the name of the Collective to reflect these practices and felt the meaning of *Wisahkotewinowak* was significant as it is an Anishinaabe word given to the Métis which describes: “the first green shoots from Mother Earth after a fire has gone through the Land.” He explained:

*I wanted to use that name in some way to reflect what we we’re doing related to the garden because I feel like in the city, in the urban context, we were trying to do something new. We were trying to figure out how to develop our land-based knowledge in a new way.*

Therefore, Land-based knowledge is being strengthened in urban centers through IFS initiatives that support relationship building to Land and to community. Access to Land enables these processes to foster Land-based responsibilities and relationships within urban centers affecting the social and physical dimensions that support healthy urban environments.

## 4. Discussion

This study was informed by Indigenous conceptions of place to explore the interactions between place and urban IFS initiatives. The thematic analysis of participant interviews generated two overarching themes: Land access, and place-making practices. Urban IFS initiatives involve Land-based activities such as sowing seeds and harvesting crops which create spaces for responsibilities, relationships, and knowledge to be formed between people and the Land-practices of place-making. Place is shaped by these interactions, and informs how IFS initiatives are taken up within urban environments. The conceptual model presented earlier in Figure 1, demonstrates the distinct, but interdependent pathways in which place interacts with IFS initiatives. 

The impacts of Land access on IFS initiatives were discussed by participants who described how relationships with landowners, landowner control and external factors affected how they were able to access and use Land for IFS initiatives. Individual and pre-existing relationships with landowners supported participants’ ability to access Land for IFS. However, structural determinants, namely processes of settler colonialism, constrained participants’ ability to access Land and pursue IFS initiatives in a fully self-determined way. Access to Land was granted with permissions, or conditional yearly arrangements demonstrating that landowners still held ultimate control and decision-making authority. Participants recognized the challenges and constraints imposed by colonial structures, which restrict self-determination to be responsible to the Land. Therefore, these structural determinants and relationships shaped by them, constitute place and impact how IFS initiatives are implemented in urban environments. 

The uptake of IFS initiatives within urban environments also generated spaces for relationship, responsibilities, and knowledge to be formed with Land and community, creating the pathway towards place-making. Participants described how carrying out Land and food-based activities such as planting seeds, harvesting, processing, and sharing food fostered responsibilities, relationships and knowledge with Land and community [45]. These processes of doing enable knowing and being, and this relational engagement with place provides crucial foundation of knowledge production [9]. Urban IFS initiatives fostered a sense of place in urban environments through place-making practices: bringing attention to the reciprocal relationships with Land, cultivating Land-based knowledge, and fostering an awareness to the responsibilities of Land and community. 

There is a paucity of literature that examines how Land access and structural determinants affect IFS initiatives, but when it is discussed is often described with challenges and limitations. For example, in the community of Kahnawà:ke, Delormier et al. [22] describe how planting on common lands within the reserve without formal permissions created a contentious political space that limited participation from the community, despite the intent to cultivate food for the benefit of the community. In an urban example, Peach et al. [44] discuss how participants were able to demonstrate self-determination by asserting control over Land use and growing processes at the Indigenous Medicine Garden at Western University, but addressed that power imbalances between garden participants and the institution prevented any decolonization goals to occur. These examples demonstrate that gaining access to Land in and of itself is not a clear path towards secured Indigenous self-determination when colonial structures govern relationships with Land. 

Colonial structures further the challenge towards self-determination within urban spaces. Primarily, cities are often overlooked as Indigenous spaces, as they were key in the colonial processes to facilitate national expansion and industrialization [37,65]. This has resulted in the erasure of urban Indigenous Peoples from policies and programs and therefore a lack of recognition for any responsibility or jurisdiction of urban Lands [37]. Participants in this study recognized these challenges, and considered how cities exist on treaty territories, adding layers of complexity as to how to move forward on these issues. Daigle [5] poses important questions to be considered: “how do Indigenous Peoples from a vast number of Nations interact with each other, and with the Indigenous laws of the Lands in which they live on in such contexts?” (p. 312).

IFS initiatives provide a starting point to explore and address these challenges, by creating spaces for community members to come into relationship with the Land and each other through the growing, harvesting, processing, and sharing of food. The IFS initiatives involved in this study all involved gardening, which is reflective of what is common in other urban and southern communities [43,44,66]. Gardens provide an initial connection to Land that elicits opportunity for programming activities and participation in Land and food-based practices. For example, the Tsartlip First Nation Garden project provided a physical space for community members to foster a meaningful relationship to community as well as ancestral Lands through the planting, harvesting and preparation of food [6]. Therefore, IFS initiatives create physical spaces on the Land (in this study, exclusively through gardens), inviting community members into relationships that are animated through programs and activities. The active participation in IFS initiatives and strengthening of relationships with Land and community demonstrates place-making. 

IFS initiatives provide pathways for place-making by creating space for relationships with Land to be forged, facilitating knowledge formation, and fostering an understanding of responsibilities. Through cultivating culturally significant seeds to the urban territories where these gardens were situated, or to their own identities, participants were able to give meaning to these urban places, instilling responsibilities to the Land and community. Responsibilities are at the core of food sovereignty to ensure food security, which is understood and carried on through ceremonies to respect the spiritual aspects of seed keeping, planting, and caring for the Land [22,67]. The transmission of Indigenous knowledge has been tied to the participation in Land and food-based practices and is further strengthened through community participation and programming [20,22,23,26]. Participation in IFS initiatives facilitates place-based knowledge and generates an awareness of place-based responsibilities. Therefore, IFS initiatives support place-making by strengthening relationships with Land in urban environments, revitalizing Indigenous knowledge, culture, and foodways. 

Fostering relationships with Land is a starting point towards IFS and Indigenous self-determination. Richmond et al. [14] considers that IFS requires a return to the cultural values and ways of knowing about food, which can be achieved through processes of environmental repossession. The concept of environmental repossession is rooted in the idea that Indigenous Peoples’ health, ways of living, and Indigenous knowledge systems are highly dependent on access to Land [14,68]. Environmental repossession takes on the importance of revitalizing connections and relationships with places through social, cultural, economic, and political processes [68]. Within urban institutional settings, social relationships enable Indigenous cultural spaces to become places of health and healing through the care of practitioners and roles of community members [69]. Gardens can serve as an important site for environmental repossession in urban areas, offering a connection to Land where sharing and learning can occur [44]. Therefore, participants in this study demonstrate processes of environmental repossession by participating in Land and food-based practices. It was through social relationships and the connection to Land which generated Land-based knowledge, and responsibilities, ultimately supporting community health and wellness. 

Environmental repossession provides a framework that could support urban Indigenous communities pursuing IFS initiatives, recognizing the importance of relationships and responsibilities regarding Land access processes. Nightingale and Richmond [70] explain how reclaiming access to Land alone is not sufficient for repossession efforts; physical spaces must foster relationships to give places meaning. These authors suggest that place-making through community building activities can facilitate relational and emotional attachments to a place [68,69]. Hatala et al. [8] argue that “environmental repossession be recast as place-making and meaning-making processes whereby people become actively engaged and entangled in shifting material and spiritual relations to ‘land’ and ‘nature’” (p. 124). Nonetheless, concepts of environmental repossession and place-making explain processes of connecting with the Land which generates relationship, meaning, significance, and additionally knowledge and responsibility to a place. 

This study demonstrates how IFS initiatives can support Indigenous self-determination and cultural resurgence within urban environments, showing what is possible in other urban contexts. Fostering a sense of place within the city challenges assumptions that “Land” can only be accessed in rural, remote, and reserve contexts [8]. Hatala et al. [8] explain how urban Indigenous youth decolonize the boundedness of place, identity and nature through reciprocity, spirituality, and sentience to characterize human-nature relationships through processes of place-making. Similarly, participants in this study show that relationships to the Land can be forged through place-making efforts facilitated by IFS initiatives. These processes have potential to create alternative pathways towards Land access and reclamation, contesting cities as colonial spaces, and asserting urban environments as places for Indigenous resurgence. By upholding Land-based responsibilities through daily actions, Indigenous Peoples enact their self-determination and connection to place [5]. Furthermore, emphasizing responsibilities to Land within urban contexts can support IFS, and more significantly, these processes can transform relationships to place that are generative for the Land and to the community that constitutes and shapes urban environments.

### 4.1. Recommendations for Future Research

Continued research in this area should understand how processes of environmental repossession can occur through IFS initiatives and other place-making efforts inclusive of policy and programming supportive of community-led initiatives. Future research can explore and advocate for Indigenous-led Land arrangements within urban environments. There is a shift emerging towards Indigenous Land governance and for settler governments to explore what Land access means from within Indigenous worldviews. The most prominent example being Indigenous Protection and Conservation Areas (IPCAs) which are being supported by the federal government. IPCAs are Lands and waters that Indigenous governments have the primary role in conserving and protecting through inherent laws, governance systems, and knowledge systems [71]. Although this means that they vary due to the diversity of Indigenous lifeways, IPCA’s share three essential elements: “They are Indigenous led; represent a long-term commitment to conservation; and elevate Indigenous rights and responsibilities” [71] (p. 36). While these protections are being more widely adopted in northern and remote contexts with First Nations communities, further research could explore whether this model could be applicable within urban contexts, governed by urban Indigenous Peoples. As the majority of Indigenous Peoples within Canada now live in urban centers [28], exploring alternative governance processes and policies are vital to have at this point in time as they can support processes of environmental repossession, and place-making. 

There are “innovative” approaches some urban Indigenous communities are pursuing to gain recognition and advocate for Land access by establishing structures which promote food sovereignty and self-determination. For example, the Sogorea Te’ Land Trust is an urban Indigenous women-led Land Trust based in the San Francisco Bay area of California that facilitates the return of Indigenous Lands to Indigenous Peoples by focusing on rematriation, cultural revitalization and Land restoration [72]. In 2017, a cultural easement was created in partnership with a food-justice nonprofit to transfer 2.5 acres of land to full title for the Sogorea Te’ Land Trust [73]. Within Canada, the formation of Indigenous Land Trusts is just beginning, but show a potential avenue for urban Indigenous Peoples to advocate for Land access. Further research will be necessary to understand how these structures can support Land access within colonial systems while upholding Indigenous worldviews, principles, and values. Perhaps Land Trusts can provide an innovative way to uphold the tenets of sharing, respect, peace, and friendship embedded in the Dish with One Spoon Wampum and Two Row Wampum, both treaties that govern the territory in southern Ontario. Ultimately, policies and governance systems which honor reciprocal relationships to Land can protect and enable urban Indigenous People’s ability to uphold food sovereignty and assert self-determination. 

### 4.2. Strengths and Limitations

The key findings from this study offer novel insight regarding Land access for urban Indigenous self-determination, and how IFS initiatives prove to be a pathway towards this by fostering place-based responsibilities, relationships, and knowledges. While the findings are contextual to the urban communities featured in this study, this research is relevant and applicable to other urban Indigenous communities facing similar goals and challenges. As well, these findings can inform processes for municipal and regional governments, institutions, organizations, and the public looking to show solidarity with Indigenous communities and support priorities around Land access and food sovereignty. Further, this paper offers considerations for new or re-imagined relationships with Land in urban environments to strengthen urban Indigenous self-determination and governance processes. 

The community-based nature of this study provides deep contextual information for the community where this research took place, but limits the generalizability of findings to other similar contexts in urban settings. The IFS initiatives included in this research were exclusively gardening and agriculture-based initiatives, but Indigenous food systems encompass a wider variety of food acquisition and processing techniques that are distinctive of place. Urban centers in coastal regions would likely have a greater emphasis on relation not just to Land, but Waters as well. The specific findings from this study offer insight into the experiences of urban Indigenous communities pursuing IFS initiatives and accessing Land, but applications of findings must be considered within the unique relationships with Land held within diverse places and contexts.

## 5. Conclusions

Overall, this study demonstrates how urban IFS initiatives facilitate place-making practices which supports Indigenous self-determination and sovereignty even within colonial spaces and constraints. Colonial structures restrict and reduce relationships with Land to be one of property, and a resource to control [74], contrasting diverse Indigenous perspectives which understand the Land to be alive, has spirit, and agency [1,3,4]. Structural determinants, largely colonial processes, create a challenging environment for IFS initiatives to access Land, by perpetuating the pervasive notion of Land ownership, but this presents a necessary paradigm shift to reimagine Land access from a place of ownership and control to one of relationship and responsibility. IFS initiatives presented a pathway to shift this framework within the local urban context by focusing on building relational responsibilities with the Land. Participants demonstrated how IFS initiatives enabled relationships with Land to be fostered, responsibilities to be upheld, and knowledge cultivated-creating a self-determined place. 

This study offers an example for other urban Indigenous communities and allied partners to strengthen relationships with Land and a sense of place in urban communities. We reflect the offering of Barker and Pickerill [7], “place shapes human action and is shaped by it in a relational way that underpins Indigenous knowledge and practices” (p. 649). These dynamic interactions offer that colonization has deeply impacted urban places, beyond this local context but in turn, new relationships and knowledge can be generated through the uptake of IFS initiatives among broader audiences to create healthy environments. Urban IFS initiatives have the potential to propel place-making in urban environments as the vehicle to engage diverse communities, organizations, and regional governments. The community-based approach of this research and application of the USAI Research Framework demonstrates the importance of participatory engagement and inter-relationality to prioritize goals and perspectives among urban Indigenous communities advancing IFS initiatives. Therefore, participatory with IFS initiatives could help foster learning and education for diverse audiences that operate within colonial structures, but are willing to shift perspectives and processes. Through participatory action and community collaboration, IFS initiatives offer a means to explore these realities by challenging colonial systems and prioritizing Indigenous principles and governance structures which are supportive and nourishing to all life within urban environments. 

## Figures and Tables

**Table 1:** Description of urban IFS initiatives and participant details.

Urban IFS Initiative	Participant Name or Pseudonym	Indigenous Identity	Locations	Land Access Arrangement(s)
Wisahkotewinowak	Dave	Métis	Kitchener, Waterloo, Guelph, Cambridge	Post-secondary institutions, schoolboard, and a non-profit organization
	Garrison	First Nations		
	Sarina	Métis		
Waterloo Region Indigenous Food Sovereignty Collective	Rachel	First Nations	Kitchener, Waterloo	Private land (residential)
	Beth	First Nations		
Waterloo Indigenous Student Centre	Lori	Cree-Métis	Waterloo	Post-secondary institution
North End Harvest Market	Nookomis	First Nations	Guelph	None

**Table 2:** Thematic categories and themes with representative quotes.

Thematic Categories	Themes	Representative Quotes
Land Access	Landowner relationships	“It took three years of asking questions and stuff like that before we were able to get [access to that land].”
	Landowner control	“The biggest barrier to food sovereignty for Indigenous people in urban centres is control of land… [I] f you don’t own land then we’re just borrowing land to grow food on, or to hunt food on. So, you can’t be sovereign right?”
	External factors	“Somebody ripped all our signs down and took all the strawberries”
Place-making Practices	Responsibilities	“We want to be responsible for the land and we want to be responsible for the community, right? ‘Cause our understanding of community comes from the land”
	Relationships	“I am growing Lenape squash, which is from my people from the East Coast ... but that is still brought here and talked about in relationships to, with me and this place, in the urban centre.”
	Knowledge	“[My dad and my grandpa] would take me into the woods and show me ‘this is this, this is how you use it, this one’s medicine’ but I didn’t really associate that as traditional food until we started doing the [food sovereignty] stuff.”

## Data Availability

The data are not publicly available due to privacy.
